# How Social Support Impact Teachers’ Mental Health Literacy: A Chain Mediation Model

**DOI:** 10.3389/fpsyg.2022.851332

**Published:** 2022-03-17

**Authors:** Sihui Li, Youyu Sheng, Yumei Jing

**Affiliations:** ^1^College of Education Sciences, Hubei Normal University, Huangshi, China; ^2^Institute of Psychology, Chinese Academy of Sciences, Beijing, China; ^3^Mental Health Education and Counselling Centre, Hubei Normal University, Huangshi, China

**Keywords:** mental health literacy, social support, coping tendency, life satisfaction, chain mediation

## Abstract

Teachers have an important social role, and their mental health literacy is very important to their own abilities as educators and to the growth and development of those they educate. This study explored the mechanism underlying the influence of social support on teachers’ mental health literacy by conducting a questionnaire survey of 573 teachers. The results showed that social support can influence teachers’ mental health literacy not only through the separate effects of life satisfaction and coping tendency but also through the chain mediation effect of life satisfaction and coping tendency; however, the direct effect of social support on the teachers’ mental health literacy is not significant. This study is conducive to understanding the internal mechanism underlying the relationship between social support and mental health literacy. It reminded us that when formulating mental health literacy promotion programs for teachers, we should not only provide adequate social support to improve but also should pay attention to improvements in their coping tendencies and life satisfaction.

## Introduction

Mental health, which has been become an important issue for a long time (American Psychiatric Association [APA], 2000; World Health Organization [WHO], 2004). Previous studies have been found that schools can play an important role in the promotion of positive mental health (UCLA School Mental Health Project: Center for Mental Health in Schools, 2009; Wei and Kutcher, 2012). At the same time, it has been found that one of the most effective ways to improve the schools’ role is to improve teachers’ mental health literacy. For example, Miller et al. (2019) have been found that improving the teacher’s mental health literacy can have an indirect impact on students, which in turn is helpful for the effective implementation of mental health promotion programs (Whitley et al., 2018). Intervention studies with teachers as the focus have found that, after teachers receive mental health literacy training, their mental health knowledge increases, which enhances their confidence in providing help to others and makes them more likely to take the lead in early intervention actions to students (Mazzer and Rickwood, 2015). Therefore, this study aims to explore the factors that influence teachers’ mental health literacy and the mechanism underlying it to lay a solid foundation for the development of a more complete plan for improving teachers’ mental health literacy.

### Relationship Between Social Support and Teachers’ Mental Health Literacy

Mental health literacy refers to knowledge and beliefs that help individuals identify, manage, and prevent mental illness, including the following six factors: the ability to identify specific disorders and different types of psychological distress; knowledge and beliefs about risk factors and causes; knowledge and beliefs about self-help interventions; knowledge and beliefs about available professional help; mental health stigma; and information about how to seek help for mental health (Jorm, 2000). More recently, some scholars have further developed a definition of mental health literacy as “the knowledge, attitudes and habits developed by individuals to promote the mental health of themselves and others and to cope with mental illnesses in themselves and others” (Jiang et al., 2020, p. 235).

As a social and cultural environmental factor, teachers’ social support may have an impact on their mental health literacy. Social support refers to the material, emotional, informational, and instrumental assistance that individuals perceive from their social networks (Cobb, 1976). Police officers serving as peer-support team members have indicated that peer support increased their mental health knowledge and greatly reduced their sense of stigma (Milliard, 2020), thereby improving their level of mental health literacy. The main effect model of social support purports that, regardless of the stress that an individual experiences, social support plays a direct role in promoting mental health; that is, social support has a general beneficial effect, and increasing social support can effectively improve an individual’s mental health (Cohen and Wills, 1985). Compared with teachers with low levels of social support, teachers with a stable social support system have stronger self-coping abilities, a more optimistic assessment of the severity of their own and students’ problems, and stronger mental health help-seeking intentions (Andoh–Arthur et al., 2015). The buffering effect model posits that social support exists as a buffer and often works via people’s internal cognitive system (Cohen and Wills, 1985); that is, social support affects the physical and mental health of individuals in a state of stress by reducing stress. Teachers with high total scores for social support can make full use of social resources under stressful conditions and have stronger mental health help-seeking intentions and intentions to help others (Ju et al., 2015). In addition, some studies have found that individuals are more likely to receive professional psychological treatment and services when they have the support and encouragement of close partners (Woodward et al., 2008; Downs and Eisenberg, 2012). Based on these theories and empirical findings, we hypothesize that H1: Social support can have an impact on teachers’ mental health literacy.

### The Relationships Among Life Satisfaction, Social Support, and Mental Health Literacy

Life satisfaction, as a cognitive component of well-being, is an individual’s overall evaluation of his or her quality of life (Singh and Jha, 2008). Previous studies have found that social support can significantly positively predict life satisfaction; that is, the more the social support an individual receives from close friends, the more likely that individual is to express positive emotions, and the higher his or her life satisfaction is (Siedlecki et al., 2014; Kalaitzaki et al., 2021). Additionally, people with high self-esteem have a positive attitude toward seeking professional help, are more tolerant of mental health stigma, are more confident in the effects of psychological counseling and treatment (Salmivalli, 2001; Peng and Hao, 2020), and have a higher level of mental health literacy. In addition, some scholars have found that parents of children with mental illness are prone to experiencing interpersonal tension, lower levels of social support, and decreased life satisfaction (Brei et al., 2015; Ginevra et al., 2018); however, they still regard seeking professional help as a threat to their self-esteem, and most of them engage in a vicious circle of using ineffective methods to cope with stress, which results in serious stigma (Gülşen and Özer, 2009; Yildirim et al., 2020) and poor mental health literacy. However, whether these results of previous literature are equally applicable to teachers remains to be investigated. Based on these, we hypothesize that H2: Life satisfaction plays a mediating role in the impact of social support on teachers’ mental health literacy.

### The Mediating Role of Coping Tendency

Coping tendency, as a mediating variable, may be the mediating factor underlying the impact of social support on teachers’ mental health literacy. Coping tendency refers to the cognitive and behavioral methods that individuals use in the face of frustration and stress (Folkman et al., 1986); it is a specific dynamic process that can affect the resolution of stress in the short term and the physical and mental health of the individual in the long term (Zhou et al., 2017). As an individual preference, coping tendency can be divided into positive coping and negative coping; an individual’s coping tendency is predicted to be stable under emergency conditions (Bouchard et al., 2004). The social support that teachers receive may have an impact on their coping tendencies. A comparative study of teachers in regular and specialized schools in France found that when teachers receive help from colleagues during work, they are likely to adopt problem-centered positive coping strategies (Boujut et al., 2016). Earlier studies of teachers in ordinary primary and secondary schools and special education teachers in China reported similar results (Shen, 2009; Minghui et al., 2018), which indicates that the impact of teachers’ social support on their coping tendencies has cross-cultural and cross-subject consistency. Coping tendency has a significant impact on mental health literacy. Positive coping can enhance the individual’s coping efficiency, enabling the individual to maintain a good attitude in the face of stressful events, solve problems through various means, and be more willing to seek help from important others and professionals. Under these conditions, the degree of mental health stigma is relatively weak (Zhou et al., 2010), and the level of mental health literacy is high. Based on these, we hypothesize that H3: Coping tendency plays a mediating role in the impact of social support on teachers’ mental health literacy.

### The Chain Mediation Effect of Life Satisfaction and Coping Tendency

As one of the most authoritative theories in the study of cognitive behavior, Theory of Reasoned Action (TRA) has been confirmed in many fields and has also been widely used in health psychology research in recent years (Jung et al., 2017). It assumes that the individual behavior can be reasonably inferred from behavioral intention to some extent, and individual behavioral intention is determined by the attitude to behavior and subjective criteria. People’s behavioral intention is a measure of people’s intention to engage in a specific behavior, while attitude is people’s positive or negative feelings about engaging in a target behavior, which is determined by the main belief of the behavior result and the estimation of the importance of the result.

Previous studies have found that high social support has been proved to produce positive attitudes, such as positive coping tendency (Zhou et al., 2020) and high life satisfaction (Jung et al., 2017). Meanwhile, life satisfaction has also been proved to further improve coping tendency (Deniz, 2006). According to this theory of TRA, mental health literacy, as a positive behavioral intention, also can be affected by these positive attitudes. Based on these, we hypothesize that H4: Life satisfaction and coping tendency have a chain mediation role in the relationship between social support and teachers’ mental health literacy.

### Overall Hypothetical Model

In order to test these hypothesizes, this study intends to use a questionnaire survey to explore the relationships between social support and teachers’ mental health literacy and the roles of life satisfaction and coping tendency in the relationship between social support and teachers’ mental health literacy. The research hypotheses are as follows: (1) social support has an impact on teachers’ mental health literacy; (2) life satisfaction plays a mediating role in the relationship between social support and teachers’ mental health literacy; (3) coping tendency plays a mediating role in the impact of social support on teachers’ mental health literacy; and (4) life satisfaction and coping tendency play a chain mediation role in the relationship between social support and teachers’ mental health literacy.

## Materials and Methods

### Subjects

This cross-sectional study took a stratified random sampling, with participating frontline teachers were recruited from seven schools (one primary school, one junior high school, two high schools, and three universities) in Hubei Province, China. This study was conducted online through a survey website. A hyperlink to the survey was sent to E-mail address of the full-time psychology teachers at selected schools, who then forwarded the questionnaire to participants. Between May and August 2020, a total of 700 questionnaires were distributed and 573 valid questionnaires were recovered, with an effective recovery rate of 81.86%. The study was reviewed and approved by Ethics Committee of Hubei Normal University. All participants signed informed consent prior to filling out the questionnaire. They were paid 10 yuan after completing the questionnaire. Among the participants, 137 were male teachers (23.9%), and 436 were female teachers (76.1%); 78 were 21–30 years old (13.6%), 228 were 31–40 years old (39.8%), 173 were 41–50 years old (30.2%), and 94 were 50–60 years old (16.4%).

### Materials

#### Social Support Rating Scale

This study used the social support rating scale (SSRS) developed by Xiao (1994), which includes three dimensions of objective support, subjective support, and utilization of support that comprise 10 questions. The higher the total social support score is, the better the respondent’s social support. The SSRS has been proven to be highly authoritative and suitable for the Chinese population. In this study, the internal consistency coefficient was 0.830.

#### Simplified Copying Style Questionnaire

The simplified copying style questionnaire (SCSQ), developed by Xie (1998). It comprises a total of 20 items, including two subscales (positive coping and negative coping), and a four-point scale of 0–3 is used for scoring. The difference between the positive coping standard score and the negative coping standard score is the coping tendency score, and the higher the coping tendency score is, the stronger the respondent’s inclination to adopt positive coping strategies. The internal consistency coefficient for this study as 0.841.

#### Satisfaction With Life Scale

The satisfaction with life scale (SWLS), developed by Diener et al. (1985) was used. The SWLS consists of five items with responses given on a 7-point Likert scale ranging from 1 (strongly disagree) to 7 (strongly agree). The higher the total score is, the higher the individual’s satisfaction with his or her current life. The internal consistency coefficient for this study was 0.882.

#### Mental Health Literacy Questionnaire

The mental health literacy questionnaire (MHLQ) developed by Jiang et al. (2020) was used. The questionnaire includes the following six subscales: (1) knowledge and concepts related to mental health; (2) knowledge and concepts related to mental illness; (3) attitudes and behavioural tendency to promote one’s own mental health; (4) attitudes and behavioural tendency to promote the mental health of others; (5) attitudes and behavioural tendencies to cope with one’s own mental illness; and (6) attitudes and behavioural tendencies to cope with the mental illness of others. These six subscales respectively address the six core components of mental health literacy. The mental health literacy questionnaire includes 60 questions; Questions 1–30 are scored as 0 or 1, and Questions 31–60 are scored using a 5-point Likert scale. To calculate the total mental health literacy score, the responses based on the 5-point Likert scale are converted to the 0–1 point scoring system. In this study, the internal consistency coefficient is 0.838.

### Statistical Processing

SPSS21.0 was used to perform general descriptive statistics and Pearson correlation analysis (two-sided test *p* < 0.05 was considered to be significantly correlated); In order to ensure the accuracy of the results, variance inflation factor (VIF) method was used for collinearity test (if VIF > 10, there is a serious collinearity problem between the variables and the corresponding variables need to be eliminated). Model 6 in the process plug-in compiled by Hayes (2017) was used for chain mediation effect analysis, and the bias correction percentile Bootstrap method was used to test the significance of the mediation effect. If the 99% confidence interval did not contain a value of 0, it was considered statistically significant (Erceg-Hurn and Mirosevich, 2008). In addition, Harman’s one-factor test was used to test for common method deviation before analyzing the data (Podsakoff et al., 2003).

## Results

### Common Method Bias Test

Because this study used self-report scales to collect data, which can lead to common method bias, the Harman single-factor method was used to include the mental health literacy, social support, coping tendency, and life satisfaction items in an exploratory factor analysis. Only 10.411% of the variance was explained by the largest factor, which is less than the critical value of 40%, it indicates that there was no significant common method bias in this study.

### Correlation Analysis for Social Support, Coping Tendency, Life Satisfaction, and Mental Health Literacy

Pearson’s product moment correlation analysis was used to analyse social support, coping tendency, life satisfaction, and mental health literacy (Table 1). The results showed that ➀ social support is significantly positively correlated with teachers’ mental health literacy, coping tendency, and life satisfaction (*r* = 0.221, *p* < 0.01; *r* = 0.393, *p* < 0.01; *r* = 0.446, *p* < 0.01); ➁ coping tendency is significantly positively correlated with teachers’ mental health literacy and life satisfaction (*r* = 0.283, *p* < 0.01; *r* = 0.299, *p* < 0.01); ➂ life satisfaction is significantly positively correlated with teachers’ mental health literacy (*r* = 0.214, *p* < 0.01).

**TABLE 1:** Descriptive statistics and correlation matrix for each variable.

Variable	Mean (M)	Standard deviation (SD)	1	2	3	4
Mental health literacy	0.673	0.127	1			
Social support	4.593	0.795	0.221**	1		
Life satisfaction	4.526	1.358	0.214**	0.446**	1	
Coping tendency	−0.001	1.209	0.283**	0.393**	0.299**	1

**Significant correlation at the 0.01 level (Two-tailed test), p < 0.01.

### Relationship Between Social Support and Mental Health Literacy: A Chain Mediation Effect Test

The above analysis showed that there was a significant correlation between the variables and that collinearity may exist. Therefore, before testing the effect, the predictive variables in the equation were standardized, and collinearity diagnostics were performed. The results showed that the variance inflation factors (1.375, 1.277, and 1.210) of all of the predictors were less than five. Therefore, there was no serious collinearity in the data used for this study, indicating that they were suitable for further mediation effect tests.

**FIGURE 1:**
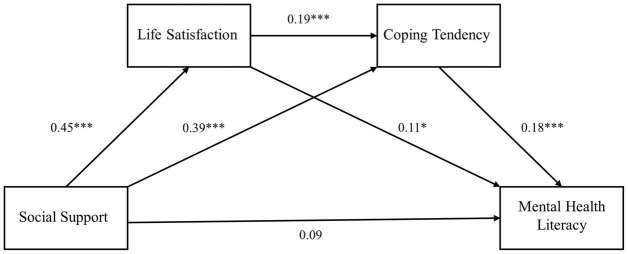
The chain mediation model. *p < 0.05 and ***p < 0.001.

The process plug-in developed by Hayes was used to evaluate the 95% confidence interval (CI) of the mediation effect of life satisfaction and coping tendency on the impact of social support on the teachers’ mental health literacy (the bootstrap sample size was 5,000), and the chain mediation model was established (Figure 1). The results showed that the predictive effect of social support on mental health literacy was not significant (β = 0.09, *p* > 0.05), but social support could significantly positively predict coping tendency and life satisfaction (β = 0.45, *p* < 0.001; β = 0.39, *p* < 0.001); life satisfaction could not only significantly predict the teachers’ coping tendency (β = 0.19, *p* < 0.001) but could positively predict their mental health literacy (β = 0.11, *p* < 0.05); and coping tendency could significantly positively predict the teachers’ mental health literacy (β = 0.18, *p* < 0.001).

Further testing of the mediation effect (see the Table 2) showed that the bootstrap 95% CI of the total effect of life satisfaction and coping tendency on the impact of social support on the teachers’ mental health literacy was (0.0812, 0.1893), which does not include 0; this indicated that life satisfaction and coping tendency are the mediating variables in the impact of social support on teachers’ mental health literacy, and they have a total indirect effect of 0.134, accounting for 60.56% of the total effect. This mediation effect is mainly composed of the following three paths: (1) social support→life satisfaction→mental health literacy [95% CI = (0.0084, 0.0911), standard error (SE) = 0.0207], the mediation effect is 0.0494, accounting for 22.32% of the total effect, and Hypothesis 1 is supported; (2) social support→coping tendency→mental health literacy [95% CI = (0.0387, 0.1065), SE = 0.0174], the mediation effect is 0.0698, accounting for 31.54% of the total effect, and Hypothesis 2 is supported; (3) social support→life satisfaction→coping tendency→mental health literacy [95% CI = (0.0055, 0.0266), SE = 0.0054], the mediation effect is 0.0148, accounting for 6.69% of the total effect, and Hypothesis 3 is supported.

**TABLE 2:** Bootstrap analysis of the mediation effect test.

	Effect	Boot SE	Boot LL CI	Boot UL CI	Relative mediation effect
Indirect effect 1	0.0494	0.0207	0.0084	0.0911	22.32%
Indirect effect 2	0.0698	0.0174	0.0387	0.1065	31.54%
Indirect effect 3	0.0148	0.0054	0.0055	0.0266	6.69%

## Discussion

This study explores the effects of different social supports on teachers’ mental health literacy and the mediating role of life satisfaction and coping tendency. The results show that social support is significantly positively correlated with teachers’ mental health literacy, life satisfaction, and coping tendency; social support can not only influence teachers’ mental health literacy through life satisfaction and coping tendency separately but can also indirectly influence teachers’ mental health literacy through the chain mediation effect of life satisfaction and coping tendency.

First, life satisfaction mediates the impact of social support on teachers’ mental health literacy; that is, obtaining social support can improve individuals’ life satisfaction and thus promote the improvement of their mental health literacy. The results show that the direct effect of social support on the teachers’ mental health literacy is not significant, which is inconsistent with the results of previous studies, i.e., that individuals’ mental health literacy can be improved through peer support (Kola-Palmer et al., 2020). That is, there is a snowball effect of seeking professional help: when an individual seeks professional help, private interpersonal conversations can result in changes in the attitudes of those around him/her toward seeking help, which could promote their own help-seeking behavior. This finding may relate to common mental health problems. Teachers with poor mental health who receive social support from parents or friends may reduce their pain in the short term when faced with common mental health problems (such as depression) and greatly alleviate their discomfort, so they largely believe that they no longer need to seek formal mental health services (Clark et al., 2020). Robichaud and Dugas (2012) also came to the same conclusion in the generalized anxiety disorder population, that regular reassurance (often from parents in adolescents) can reduce short-term suffering, although eventually the anxiety disorder remains. Based on the buffering effect model (Cohen and Wills, 1985), the influence of the external environment (such as social support) plays a role via the individual’s personal factors (such as life satisfaction). Teachers who receive sufficient social support have a higher level of understanding and use of social support resources, and experience higher life satisfaction after experiencing more positive emotions, which can reduce stress in the face of stressful events, have a more positive attitude toward seeking professional help, have a strong willingness to help when they suspect that others are having psychological problems, and have a high mental health literacy. In contrast, teachers who lack social support during their careers can have reduced life satisfaction, and the lack of happiness and weak psychological capital can easily make it difficult for them to extricate themselves from negative emotions (Huang et al., 2020). Because of their own pessimism and negativity, teachers with a low level of social support are more likely to have negative views regarding professional help and treatment effects, which seriously affects their mental health literacy.

Second, this study shows that coping tendency has a mediating role in the relationship between social support and teachers’ mental health literacy. The results of this study are consistent with previous studies (Dong, 2019). Research shows that social support can significantly positively predict coping strategies (Kong et al., 2019). Teachers with a high level of social support have stronger social adaptation abilities and tend to adopt positive coping strategies for solving problems. When their mental state is abnormal, they are also more willing to help themselves, seek help from important others and professionals, and have a low sense of stigma regarding psychological counseling and treatment. In addition, due to the psychology of repayment, social support is significantly related to altruistic behavior. Individuals who receive social support are prone to show altruistic behavior (Romig and Bakken, 1992), and they can often identify and help others’ mental health problems in a timely manner based on their own experience.

Finally, this study shows that life satisfaction and coping tendency separately could predict teachers’ mental health literacy. Based on the TRA (Fishbein and Ajzen, 1977), positive behavioral intention, can be affected by these positive attitudes. Teachers with high life satisfaction, or positive coping tendency, can clearly understand themselves and make full use of their own resources, and can actively self-regulate to eliminate external pressure. These positive attitudes allow teachers to have positive behavioral intention, regarding the prevention and treatment of possible psychological problems, which effectively improves their mental health literacy.

As a relatively important type of social group, the mental health status of teachers is crucial to their own ability to educate people and the growth and development of the educated group, and improving the mental health literacy of teachers can expand the beneficiary group to all students, which suggests that we should pay more attention to the mental health literacy of the majority of teachers. This study explores the possible influencing factors of teachers’ mental health literacy, fills the gap in the literature to a certain extent and helps us to deeply understand the mechanism of life satisfaction and coping tendencies between social support and teachers’ mental health literacy, and provides possible directions for future plans to improve teachers’ mental health literacy.

However, the current study still has some limits. First, this study only involves the attitude and habit level of mental health literacy, there is still a lack of research on the knowledge level of mental health literacy, and a comprehensive investigation of this aspect still needs to be added in the future; Second, due to conditions, this study only studies the relationship between social support, life satisfaction, coping tendencies and teachers’ mental health literacy, and the causal relationship between various variables is not yet known, and it still needs to be further tracked and verified.

## Conclusion

1) Teachers’ social support is significantly positively correlated with their mental health literacy, coping tendency, and life satisfaction; coping tendency is significantly positively correlated with mental health literacy and life satisfaction; and life satisfaction is significantly positively correlated with mental health literacy.
2) Teachers’ social support affects mental health literacy through the separate mediation effects of life satisfaction and coping tendency.
3) Teachers’ social support indirectly affects mental health literacy through the chain mediation effect of life satisfaction and coping tendency.

## Data Availability Statement

The raw data supporting the conclusions of this article will be made available by the authors, without undue reservation.

## Ethics Statement

The studies involving human participants were reviewed and approved by Ethics Committee of Hubei Normal University. The patients/participants provided their written informed consent to participate in this study.

## Author Contributions

YJ contributed to conception and design of the study. SL and YS wrote the first draft of the manuscript. YS revised and delivered the manuscript. All authors contributed to manuscript revision, read, and approved the submitted version.

## Conflict of Interest

The authors declare that the research was conducted in the absence of any commercial or financial relationships that could be construed as a potential conflict of interest.

## Publisher’s Note

All claims expressed in this article are solely those of the authors and do not necessarily represent those of their affiliated organizations, or those of the publisher, the editors and the reviewers. Any product that may be evaluated in this article, or claim that may be made by its manufacturer, is not guaranteed or endorsed by the publisher.

## References

[B1] American Psychiatric Association [APA] (2000). *Diagnostic and Statistical Manual of Mental Disorders*, Text Revision (DSM-IV-TR), 4th Edn. Washington, DC: American Psychiatric Association.

[B2] Andoh–Arthur J. Asante K. O. Osafo J. (2015). Determinants of psychological help-seeking intentions of university students in Ghana. *Int. J. Adv. Couns.* 37 330–345. 10.1007/s10447-015-9247-2

[B3] Bouchard G. Guillemette A. Landry-Léger N. (2004). Situational and dispositional coping: an examination of their relation to personality, cognitive appraisals, and psychological distress. *Eur. J. Pers.* 18 221–238. 10.1002/per.512

[B4] Boujut E. Dean A. Grouselle A. Cappe E. (2016). Comparative study of teachers in regular schools and teachers in specialized schools in France, working with students with an autism spectrum disorder: stress, social support, coping strategies and burnout. *J. Autism Dev. Disord.* 46 2874–2889. 10.1007/s10803-016-2833-2 27282858

[B5] Brei N. G. Schwarz G. N. Klein-Tasman B. P. (2015). Predictors of parenting stress in children referred for an autism spectrum disorder diagnostic evaluation. *J. Dev. Phys. Disabil.* 27 617–635. 10.1007/s10882-015-9439-z

[B6] Clark L. H. Hudson J. L. Rapee R. M. Grasby K. L. (2020). Investigating the impact of masculinity on the relationship between anxiety specific mental health literacy and mental health help-seeking in adolescent males. *J. Anxiety Disord.* 76:102292. 10.1016/j.janxdis.2020.102292 33010663

[B7] Cobb S. (1976). Social support as a moderator of life stress. *Psychosom. Med.* 38 300–314. 10.1097/00006842-197609000-00003 981490

[B8] Cohen S. Wills T. A. (1985). Stress, social support, and the buffering hypothesis. *Psychol. Bull.* 98 310–357. 10.1037/0033-2909.98.2.310 3901065

[B9] Deniz M. E. (2006). The relationships among coping with stress, life satisfaction, decision-making styles and decision self-esteem: an investigation with turkish university students. *Soc. Behav. Pers. Int. J.* 34 1161–1170. 10.2224/sbp.2006.34.9.1161

[B10] Diener E. D. Emmons R. A. Larsen R. J. Griffin S. (1985). The satisfaction with life scale. *J. Pers. Assess.* 49 71–75. 10.1207/s15327752jpa4901_13 16367493

[B11] Dong Y. (2019). *The Influence and Improvement of Social Support and Coping Tendency on the Mental Health of Primary and Secondary School Teachers: The Mediating Role of Mental Health Literacy.* (Ph.D Thesis) (Jilin City: Beihua University)

[B12] Downs M. F. Eisenberg D. (2012). Help seeking and treatment use among suicidal college students. *J. Am. Coll. Health.* 60 104–114. 10.1080/07448481.2011.619611 22316407

[B13] Erceg-Hurn D. M. Mirosevich V. M. (2008). Modern robust statistical methods: an easy way to maximize the accuracy and power of your research. *Am. Psychol.* 63 591–601. 10.1037/0003-066X.63.7.591 18855490

[B14] Fishbein M. Ajzen I. (1977). Belief, attitude, intention and behaviour: an introduction to theory and research. addison-wesley, reading ma. *Philos. Rhetoric.* 41 842–844. 10.2307/4393175

[B15] Folkman S. Lazarus R. S. Gruen R. J. DeLongis A. (1986). Appraisal, coping, health status, and psychological symptoms. *J. Pers. Soc. Psychol.* 50 571–579. 10.1037//0022-3514.50.3.571 3701593

[B16] Ginevra M. C. Di Maggio I. Santilli S. Sgaramella T. M. Nota L. Soresi S. (2018). Career adaptability, resilience, and life satisfaction: a mediational analysis in a sample of parents of children with mild intellectual disability. *J. Intellect. Dev. Disabil.* 43 473–482. 10.3109/13668250.2017.1293236

[B17] Gülşen B. Özer F. (2009). Families’ status of coping with stress who have a handicapped child. Türk Sİlahlı Kuvvetlerİ Koruyucu Hekİm. *Bül.* 8 413–420.

[B18] Hayes A. F. (2017). *Introduction to Mediation, Moderation, and Conditional Process Analysis: a Regression-Based Approach.* New York: Guilford publications.

[B19] Huang Z. Yu M. Wang J. Hu T. Zheng Y. (2020). The Relationship Between Professional Psychological Help-Seeking Attitudes and Social Support in medical colleges Students: Mediation Effect of Self-Esteem. *J. Int. Psych.* 47 312–315+321.

[B20] Jiang G. Zhao C. Wei H. Yu L. Li D. Lin X. (2020). Mental health literacy: connotation, measurement and new framework. *J. Psychol. Sci.* 43 232–238.

[B21] Jorm A. F. (2000). Mental health literacy: public knowledge and beliefs about mental disorders. *Br. J. Psychiatry.* 177 396–401. 10.1192/bjp.177.5.396 11059991

[B22] Ju C. Lan J. Li Y. Feng W. You X. (2015). The mediating role of workplace social support on the relationship between trait emotional intelligence and teacher burnout. *Teach. Teach. Educ.* 51 58–67. 10.1016/j.tate.2015.06.001

[B23] Jung H. von Sternberg K. Davis K. (2017). The impact of mental health literacy, stigma, and social support on attitudes toward mental health help-seeking. *Int. J. Ment. Health Pr.* 19 252–267. 10.1080/14623730.2017.1345687

[B24] Kalaitzaki A. Tsouvelas G. Koukouli S. (2021). Social capital, social support and perceived stress in college students: the role of resilience and life satisfaction. *Stress Health* 37 454–465. 10.1002/smi.3008 33206451

[B25] Kola-Palmer S. Lewis K. Rodriguez A. Kola-Palmer D. (2020). Help-seeking for mental health issues in professional rugby league players. *Front. Psychol.* 11:570690. 10.3389/fpsyg.2020.570690 33071903 PMC7541693

[B26] Kong L. N. Zhu W. F. He S. Yao Y. Yang L. (2019). Relationships among social support, coping strategy, and depressive symptoms in older adults with diabetes. *J. Gerontol. Nurs.* 45 40–46. 10.3928/00989134-20190305-03 30917204

[B27] Mazzer K. R. Rickwood D. J. (2015). Teachers’ and coaches’ role perceptions for supporting young people’s mental health: multiple group path analyses. *Aust. J. Psychol.* 67 10–19. 10.1111/ajpy.12055

[B28] Miller L. Musci R. D’Agati D. Alfes C. Beaudry M. B. Swartz K. (2019). Teacher mental health literacy is associated with student literacy in the adolescent depression awareness program. *Sch. Ment. Health.* 11 357–363. 10.1007/s12310-018-9281-4 31579430 PMC6774623

[B29] Milliard B. (2020). Utilization and impact of peer-support programs on police officers’ mental health. *Front. Psychol.* 11:1686. 10.3389/fpsyg.2020.01686 32765375 PMC7381167

[B30] Minghui L. Lei H. Xiaomeng C. Potmìšilc M. (2018). Teacher efficacy, work engagement, and social support among Chinese special education school teachers. *Front. Psychol.* 9:648. 10.3389/fpsyg.2018.00648 29867634 PMC5949842

[B31] Peng T. Hao Z. (2020). Influence of hope on attitudes toward professional psychological help in college students: the chain mediating effect of self-esteem and stigma. *Chin. J. Clin. Psychol.* 28 1270–1273.

[B32] Podsakoff P. M. MacKenzie S. B. Lee J.-Y. Podsakoff N. P. (2003). Common method biases in behavioral research: a critical review of the literature and recommended remedies. *J. Appl Psychol.* 88 879. 10.1037/0021-9010.88.5.879 14516251

[B33] Robichaud M. Dugas M. J. (2012). *Cognitive-Behavioral Treatment for Generalized Anxiety Disorder: from Science to Practice.* New York: Routledge.

[B34] Romig C. Bakken L. (1992). Intimacy development in middle adolescence: Its relationship to gender and family cohesion and adaptability. *J. Youth Adolsence.* 21 325–338. 10.1007/BF01537021 24263846

[B35] Salmivalli C. (2001). Feeling good about oneself, being bad to others? Remarks on self-esteem, hostility, and aggressive behavior. Aggress. *Violent Behav.* 6 375–393. 10.1016/S1359-1789(00)00012-4

[B36] Shen Y. E. (2009). Relationships between self-efficacy, social support and stress coping strategies in Chinese primary and secondary school teachers. *Stress Health* 25 129–138. 10.1002/smi.1229

[B37] Siedlecki K. L. Salthouse T. A. Oishi S. Jeswani S. (2014). The relationship between social support and subjective well-being across age. *Soc. Indic. Res.* 117 561–576. 10.1007/s11205-013-0361-4 25045200 PMC4102493

[B38] Singh K. Jha S. D. (2008). Positive and negative affect, and grit as predictors of happiness and life satisfaction. *J. Indian Acad. Appl. Psychol.* 34 40–45.

[B39] UCLA School Mental Health Project: Center for Mental Health in Schools (2009). *Mental Health in Schools: Program and Policy Analysis.* Retrieved June 16, 2009, from http://smhp.psych.ucla. edu/

[B40] Wei Y. Kutcher S. (2012). “Interntional school mental health: Global approaches, global challenges and global opportunities,” in *Evidence-based school psychiatry. Child and adolescent psychiatric clinics of North America*, eds Bostic J. Bagnell A. (The Netherlands: Elsevier), 11–28. 10.1016/j.chc.2011.09.005 22137808

[B41] Whitley J. Smith J. D. Vaillancourt T. Neufeld J. (2018). “Promoting mental health literacy among educators: a critical aspect of school-based prevention and intervention,” in *Handbook of School-Based Mental Health Promotion: An Evidence-Informed Framework for Implementation*, eds Leschied A. W. Saklofske D. H. Flett G. L. (Cham: Springer International Publishing), 143–165.

[B42] Woodward A. T. Taylor R. J. Bullard K. M. Neighbors H. W. Chatters L. M. Jackson J. S. (2008). Use of professional and informal support by African Americans and Caribbean blacks with mental disorders. *Psychiatr. Serv.* 59 1292–1298. 10.1176/appi.ps.59.11.1292 18971405 PMC2955359

[B43] World Health Organization [WHO] (2004). *The Global Burden of Disease.* Geneva: WHO.

[B44] Xiao S. (1994). The theoretical basis and research application of social support rating scale. *J. Clin. Psychol. Med.* 98–100. 10.1186/s12913-016-1423-5 27409075 PMC4943498

[B45] Xie Y. (1998). The role of coping tendency in the relationship between self-esteem and self consistency & congruence: mediator or moderator? *Chin. J. Clin. Psychol.* 2 53–54.

[B46] Yildirim G. Ertekin Pinar S. Ucuk S. Duran Aksoy O. Ersan E. E. (2020). The effect of training given to parents with mentally disabled children on their life satisfaction self-stigma of seeking help depression and stress-coping styles. *Int. J. Soc. Psychiatry.* 66 279–291. 10.1177/0020764020903750 32114867

[B47] Zhou H. Liang Y. Liu X. (2020). The effect of the life satisfaction on internet addiction in college students: the multiple mediating roles of social support and self-esteem. *Chin. J. Clin. Psychol.* 28 919–923. 10.16128/j.cnki.1005-3611.2020.05.012

[B48] Zhou X. Cecilia S. Shi Q. (2010). Mental health problems, coping mechanisms and professional help-seeking attitude in medical college students. *Chin. Ment. Health J.* 24 790–795.

[B49] Zhou Y. Li D. Li X. Wang Y. Zhao L. (2017). Big five personality and adolescent Internet addiction: the mediating role of coping style. *Addict. Behav.* 64 42–48. 10.1016/j.addbeh.2016.08.009 27543833

